# Recommendations for patient screening in ultra-rare inherited metabolic diseases: what have we learned from Niemann-Pick disease type C?

**DOI:** 10.1186/s13023-018-0985-1

**Published:** 2019-01-21

**Authors:** María-Jesús Sobrido, Peter Bauer, Tom de Koning, Thomas Klopstock, Yann Nadjar, Marc C Patterson, Matthis Synofzik, Chris J Hendriksz

**Affiliations:** 1 Neurogenetics Research Group, Instituto de Investigación Sanitaria, Santiago de Compostela, Spain; 2 Insititute of Medical Genetics and Applied Genomics, Tübingen University, Tübingen, Germany; 3 CENTOGENE AG, Rostock, Germany; 4 University of Groningen, Groningen, The Netherlands; 5 Department of Neurology, Friedrich-Baur-Institute, University Hospital of the Ludwig-Maximilians-Universität München, Munich, Germany, German Center for Neurodegenerative Diseases (DZNE), Munich, Germany, and Munich Cluster for Systems Neurology (SyNergy), Munich, Germany; 6 Department of Neurology, Reference Centre for Lysosomal Diseases (CRML), UF Neurogenetics and Metabolism, Pitié-Salpêtrière Hospital, Paris, France; 7 Mayo Clinic, Rochester, MN USA; 8 Department of Neurodegenerative Diseases, Hertie-Institute for Clinical Brain Research, University of Tübingen, Tübingen, Germany; 9 German Center for Neurodegenerative Diseases (DZNE), Tübingen, Germany; 10 University of Pretoria, Pretoria, South Africa

**Keywords:** Niemann-Pick disease, Screening, Diagnosis, Ultra-rare disease

## Abstract

**Background:**

Rare and ultra-rare diseases (URDs) are often chronic and life-threatening conditions that have a profound impact on sufferers and their families, but many are notoriously difficult to detect. Niemann-Pick disease type C (NP-C) serves to illustrate the challenges, benefits and pitfalls associated with screening for ultra-rare inborn errors of metabolism (IEMs).

A comprehensive, non-systematic review of published information from NP-C screening studies was conducted, focusing on diagnostic methods and study designs that have been employed to date. As a key part of this analysis, data from both successful studies (where cases were positively identified) and unsuccessful studies (where the chosen approach failed to identify any cases) were included alongside information from our own experiences gained from the planning and execution of screening for NP-C. On this basis, best-practice recommendations for ultra-rare IEM screening are provided. Twenty-six published screening studies were identified and categorised according to study design into four groups: 1) prospective patient cohort and family-based secondary screenings (18 studies); 2) analyses of archived ‘biobank’ materials (one study); 3) medical chart review and bioinformatics data mining (five studies); and 4) newborn screening (two studies). *NPC1/NPC2* sequencing was the most common primary screening method (Sanger sequencing in eight studies and next-generation sequencing [gene panel or exome sequencing] in five studies), followed by biomarker analyses (usually oxysterols) and clinical surveillance.

**Conclusions:**

Historically, screening for NP-C has been based on single-patient studies, small case series, and targeted cohorts, but the emergence of new diagnostic methods over the last 5–10 years has provided opportunities to screen for NP-C on a larger scale. Combining clinical, biomarker and genetic diagnostic methods represents the most effective way to identify NP-C cases, while reducing the likelihood of misdiagnosis. Our recommendations are intended as a guide for planning screening protocols for ultra-rare IEMs in general.

**Electronic supplementary material:**

The online version of this article (10.1186/s13023-018-0985-1) contains supplementary material, which is available to authorized users.

## Introduction

Rare and ultra-rare diseases (URDs) are often chronic and life-threatening conditions that have a profound impact on sufferers and their families, but many are notoriously difficult to detect. Between 5000 and 8000 distinct rare diseases are documented (www.eurordis.org). Individually, these diseases are infrequent but collectively they affect 300 million people worldwide (www.eurordis.org) [1]. The definition of a URD varies based on different factors, including disease prevalence, symptom severity/impact, treatment availability, and heritability [2]. In the EU a URD is defined as affecting < 2:100,000 people (< 20 patients per million) [3, 4]. Inborn errors of metabolism (IEMs) represent a group of URDs collectively reported to affect up to 125:100,000 people [5, 6].

Ultra-rare IEMs have received increased attention in the last two decades due to the characterisation of causal genes and underlying metabolic pathways. This has enabled the development of targeted, disease-modifying treatments for a number of such conditions, including Niemann-Pick disease types A, B and C (NP-A/NP-B/NP-C), Gaucher disease type 3 (GD3), Fabry disease, phenylketonuria (PKU), medium-chain acyl-CoA dehydrogenase deficiency (MCADD) and homocysteinemia, among others [1, 7, 8]. Such therapies can have a major effect on disease course, increasing patient quality of life and improving outcomes [9–11], but early and prompt initiation of treatment is usually required to minimise or prevent irreversible pathology (e.g., neuronal damage in neurodegenerative IEMs). Proactive strategies to enable timely diagnosis are therefore essential.

NP-C is an autosomal recessive, neurovisceral lysosomal storage disease (LSD) caused by mutations in the *NPC1* or *NPC2* genes (in ~ 95% and ~ 5% of patients, respectively) [11, 12]. These lead to impaired intracellular lipid trafficking and excess glycosphingolipid storage in various tissues including the brain and liver [13]. Affected patients exhibit highly heterogeneous clinical phenotypes involving progressive neurological and psychiatric manifestations as well as visceral symptoms [11]. The disease has pan-ethnic occurrence and has been estimated to affect at least 1:100,000 individuals [1, 12, 14]. However, it is believed that the true prevalence of the disease is higher, as cases can be masked by non-specific symptoms in certain clinical subpopulations [14, 15].

NP-C serves as a prime example to illustrate the challenges, benefits and pitfalls associated with screening for an ultra-rare IEM, since it has a number of features common to most such diseases [16]. It is a chronic, progressive condition involving high clinical heterogeneity and early mortality, and often goes undetected or misdiagnosed for prolonged periods due to non-specific manifestations. Diagnosis requires multidisciplinary work-up and multiple referrals to expert centres. There is limited awareness of symptoms suggestive of NP-C at the routine practice level, which can delay specialist referral and accurate diagnostics. Table 1 summarises the key features of NP-C alongside other IEMs with similar characteristics. All of them are URDs, with variable age at onset and heterogeneous clinical phenotype, almost invariably involving diverse neuropsychiatric manifestations.

**Table 1:** Comparison of NP-C with other, similar ultra-rare IEMs

	NP-C	Tay-Sachs disease	MEGDEL syndrome	Krabbe disease	Gaucher disease type 3
Biological and biomarker features					
Genes	*NPC1/NPC2*	*HEXA*	*SERAC1*	*GALC*	*GBA*
Cellular markers	Filipin staining	–	Filipin staining, phosphatidylglycerol 34:1/36:1 levels [68, 69]	–	–
Fluid/enzymatic diagnostic biomarkers	Plasma oxysterols, bile acids, and lysosphingolipids (e.g. Lyso-SM-509)	Plasma lyso GM2 ganglioside; plasma HEXA activity	Urine 3-MGA/3-MGC	Plasma GALC activity	Plasma glucocerebrosidase activity, lyso-GB1
Clinical manifestations	Adult ataxia, VSSP, dystonia, myoclonus, dysphagia, dysarthria, cataplexy, psych. problems, visceral symptoms^a^	SEM abnormalities, dysarthria, ataxia, dystonia, lower MND, myoclonus, epilepsy, psych. problems	Hearing loss, dystonia, spasticity, dysarthria	Vision/hearing loss, ataxia, dysmetria, spasticity	Hepatosplenomegaly, thrombocytopenia, anaemia/fatigue, bone abnormalities, HSGP, ataxia, parkinsonism, epilepsy
Rarity and range of clinical features mandating a need for screening studies					
Ultra-rare	Yes	Yes	Yes	Yes	Yes
Variable phenotype, mainly neurological	Yes	Yes	Yes	Yes	Yes
Onset from infancy to adulthood	Yes	Yes	Yes [69]	Yes	Yes

^a^Other, non-specific neurological manifestations can also be present. *GALC* galactocerebrosidase, *GBA* beta-glucocerebrosidase, *HEXA* hexosaminidase A, *HSGP* horizontal supranuclear gaze palsy, *MEGDEL* 3-methylglutaconic aciduria with deafness, encephalopathy, and Leigh-like syndrome, *3-MGA* 3-methylglutaconic acid, *3-MGC* 3-methylglutaric acid, *MND* motor neurone disease, *Psych.* psychiatric, *SEM* saccadic eye movement, *SERACC1* Serine Active Site Containing protein 1, *VSSP* vertical supranuclear saccade palsy

The diagnosis of NP-C used to depend on time-consuming and costly laboratory techniques such as filipin staining and cholesterol esterification assays, with confirmatory Sanger genetic sequencing in single patients [10, 17–19]. However, increased knowledge of the disease has allowed the development of new screening and diagnostic methods. Simple clinical tools such as the NP-C suspicion Index (NP-C SI) help detect patients with a high likelihood of NP-C for further testing [20–22]. Rapid, reliable and cost-effective blood biomarkers including oxysterols [23], lysosphingomyelins [24, 25], and bile acids are also now available [18, 26]. In addition, powerful next-generation sequencing (NGS) methods, whole-exome sequencing (WES) and phenotype-specific gene panels can now be applied to entire patient cohorts as well as single patients [23, 26–28].

Disease screening can involve testing complete populations of asymptomatic individuals for the presence of certain disease markers. However, screening for ultra-rare IEMs on a population-wide basis is not generally considered appropriate due to a number of ethical, health economic, legal and regulatory limitations. Instead, ultra-rare IEMs are typically screened for through targeted testing of at-risk cohorts with certain relevant symptoms or risk factors. The WHO criteria for disease screening specify that new screening technologies must address a number of factors relevant to many URDs [29, 30]. An accepted treatment for the disease being screened must be available, the tests must be accessible, and the disease must feature a recognizable latent or early symptomatic stage, all of which are true for NP-C.

In this article, we review the wide range of methods and study designs that have been used to screen for NP-C, taking in lessons from both successful studies (where screening succeeded in identifying new cases) and unsuccessful studies (where the chosen approach failed to identify any cases). Specific ‘diagnostic methods’ include genetic testing, biomarker analysis, and clinically-based techniques. The term ‘study design’, as applied in our review, refers to overall screening approaches split into four categories: 1) prospective patient screening studies; 2) analyses of archived ‘biobank’ materials; 3) medical chart review and bioinformatics data mining; and 4) newborn screening. We reviewed the experience gained from the planning and execution of screening studies in NP-C as a representative example of an ultra-rare IEM. Finally, we propose best practice recommendations that we believe could be extrapolated to screening protocols for other rare IEMs.

## Methodology

A comprehensive, non-systematic review of published information was conducted using PubMed and Embase. All NP-C screening studies or studies in which NP-C was detected during screening of patients with unknown aetiologies were considered eligible. Articles published in English or at least with English abstracts between 2000 and 2018 were included. The main search terms were ‘Niemann’, ‘screening’ and/or ‘diagnosis’ (limited mainly to title/abstract fields). A pragmatic approach was adopted for the inclusion of articles due to the extremely varied nature of published literature relating to URD screening studies. No protocol for handling case redundancy between publications was included in the search since the emphasis of this review was on methodological approaches as opposed to establishing disease prevalence.

Each identified publication was examined to extract methodological features relating to: study population (e.g., population size, patient age, clinical phenotype); study type (e.g., observational or interventional, prospective or retrospective, controlled or not controlled/naturalistic), diagnostic methods, study location (e.g., regional/international, single-centre/multicentre), medical specialty/disease area (e.g., neurology, paediatrics, hepatology, “any”), and inclusion of controls (e.g., healthy controls, disease-area controls). Available, unpublished methodological aspects of some of our own ongoing screening studies were also described, where relevant.

All identified studies were grouped in summary Table 2 based on overall study design (screening types). Further details of the included studies are provided in Additional files 1, 2 and 3 Table S1-S3, categorised by the primary diagnostic method. Many of the studies involved a combination of clinical, biochemical and genetic methods.

**Table 2:** Summary of published screening studies grouped by screening design

Cohort (reference)	Study population	*N*	Region/country	Primary screening method(s)
Prospective patient screening studies				
*Genetic screening*				
Bauer et al. 2013 [31]	Adults with neurological/psych. Symptoms	250	EU and US	*NPC1/NPC2* sequencing^a^, gene dosage analysis
Schicks et al. 2013 [32]	Adults with ataxia, EOCD and suspected recessive disease	24	Germany	*NPC1/NPC2* sequencing^a^
Zech et al. 2013 [36]	Adults with PD, FTD or PSP	790	Germany	*HRM followed by NPC1/NPC2* sequencing^a^
Nanetti et al. 2017 [33]	Adults with suspected HD	18	Italy	*NPC1/NPC2* sequencing^a^
Topcu et al. 2017 [34]	Family members of NPC1/NPC2 probands	510	Turkey	*NPC1/NPC2* sequencing^a^
Cupidi et al. 2017 [35]	Adults with early-onset ‘dementia-plus’	50	Italy	*NPC1/NPC2* sequencing^a^, gene dosage analysis
Synofzik et al. 2015 [27]	Adolescents/adults with unexplained EOA	96	Germany	*NPC1/NPC2* (NGS gene panel^b^)
Marelli et al. 2016 [40]	Adolescents/adults with probable EOA	33	France	*NPC1/NPC2* (NGS gene panel)
Pyle et al. 2015 [41]	Adult patients with inherited and sporadic ataxias	35	UK	*NPC1/NPC2* sequencing (WES)
McKay et al. 2014 [42]	Infants with jaundice/cholestasis	228	UK	*NPC1/NPC2 sequencing* (WES)
Herbst et al. 2015 [43]	Infants with jaundice/cholestasis	6	Germany	NPC1/NPC2 sequencing (NGS gene panel)
Mavridou et al. 2014 [70]	Family members of NP-C patients	153	Greece	*NPC1/NPC2* sequencing^a^, RFLP analysis
Wassif et al. 2016 [14]	Subjects from 4 WES sequencing projects	17,754^c^	International	Historical WES + WES from public databases
*Blood biomarker screening*				
Reunert et al. 2016 [44]	Patients with suspected NP-C	1800	Germany	Oxysterol level (C-triol)
Ribas et al. 2016 [45]	Patients with suspected NP-C	122	Brazil	Oxysterol level (C-triol), ChT
Zhang et al. 2014 [46]	Children/adults with cholestasis, HSL or psychomotor regression/retardation	302	China	Oxysterol level (7-KC)
De Castro et al. 2017 [47]	Patients with suspected NP-C	236	Spain	ChT, CCL18/PARC, *NPC1/NPC2* sequencing^a^
Sheth et al. 2014 [48]	Children with possible LSDs	1110	India, Sri Lanka, Afghanistan	Urine GAGs, plasma ChT, enzyme activity
Studies based on archived (biobank) samples				
Cebolla et al. 2015 [50]	Patients with NP-C	97	Spain	Oxysterol level (7-KC)
Studies based on patient file and clinical chart review				
Yerushalmi et al. 2002 [51]	Neonates with jaundice/cholestasis	40	US	Medical chart review
Hegarty et al. 2015 [52]	Children with acute liver failure	127	UK	Clinical, laboratory, and outcome analysis
Verity et al. 2010 [53]	Children with early cognitive impairment	2636	UK	Clinical case surveillance
Winstone et al. 2017 [54]	Children with intellectual and neurological deterioration	3979	UK	Clinical case surveillance
Corry 2014 [55]	Ethnic subjects with suspected autosomal recessive conditions	13,000	UK	Clinical case surveillance
Studies based on newborn screening				
Pinto et al. 2004 [60]	Antenatal patients with suspected LSDs	353	Portugal	*NPC1/NPC2* sequencing^a^
Polo et al. 2016 [61]	Neonates with cholestasis	7	Italy	Oxysterols (7-KC, C-triol)

^a^Sanger sequencing; ^b^Mini-exome sequencing of ~5,000 genes; ^c^WES of 17,754 chromosomes. *ChT* chitotriosidase, *CNV* copy-number variation, *EOA* early-onset ataxia, *EOCD* early-onset cognitive decline, *FTD* frontotemporal dementia, *GAG* glycosaminoglycans, *HRM* high resolution melting, *HSL* hepatosplenomegaly, *LSD* lysosomal storage disease, *NGS* next-generation sequencing, *PD* Parkinson’s disease, *PSP* progressive supranuclear gaze palsy, *Psych.* psychiatric, *RFLP* restriction fragment length polymorphism, *WES* whole-exome sequencing

Findings from each published study were presented as the net number as well as the proportion (%) of NP-C patients identified. Methodological details and relevant learnings from ‘failed’ studies, in which no NP-C patients were identified, were also considered. In addition, mid- to long-term ‘halo’ effects of screening studies were addressed in order to gauge any lasting impact due to increased awareness and implementation of new methods (e.g., subsequent inclusion of NP-C in local diagnostic protocols).

## Findings

### Prospective patient screening studies

Numerous prospective NP-C screening studies narrowed the screening focus by targeting cohorts with an increased disease risk, and found patients with NP-C who had previously gone undetected. Most such studies involved combinations of initial clinical assessments with one or both of genetic and biomarker analyses.

#### Genetic screening

Historically, the most widely used genetic analysis method for confirming a diagnosis of NP-C has been Sanger sequencing of *NPC1* and *NPC2* in individual patients with symptoms that are strongly suggestive of NP-C [11, 26]. However, a number of studies also used this method to identify new cases within at-risk cohorts (Table 2; Additional file 1: Table S1). In a cohort of 250 adults with neuropsychiatric symptoms compatible with NP-C, Bauer et al. observed a higher incidence of NP-C (1.2%) versus that in the general population (1–1.12:100,000 individuals (0.001%)) [31]. In addition, 12 (4.8%) heterozygous NP-C carriers (i.e., individuals with single *NPC1/NPC2* variants) were identified. NP-C cases have also been successfully identified using direct Sanger sequencing in patients with early-onset degenerative ataxia [32] and Huntington’s disease-like manifestations (HD) [33].

Targeted Sanger-based screening of relatives following the diagnosis of probands with *NPC1/NPC2* variants confirmed a high prevalence of NP-C carriers in some regions. Based on a Turkish National Registration Database, Topcu et al. screened 510 family members of four NP-C probands with data suggestive of consanguinity. Two new NP-C patients (0.4%) from two families were identified [34]. Notably, the overall frequency of heterozygous *NPC1/NPC2* carriers in this cohort was 22.7%.

Cohort studies have also been published in which no patients were diagnosed with NP-C using Sanger sequencing. Among 50 adults with early-onset neurodegenerative dementia and atypical symptoms (‘dementia plus syndrome’), Cupidi et al. only observed four individuals with single *NPC1* or *NPC2* variants [35], and suggested a possible contributing role for *NPC1/NPC2* variants in these cases. In a large comparative cohort of patients with Parkinson’s disease (PD), frontotemporal dementia (FTD) and progressive supranuclear palsy (PSP), Zech et al. reported identified only a single pathogenic *NPC1/NPC2* variants in six patients (1.1%), which did not differ significantly from the frequency of heterozygous variants in the general population [36].

Large NGS gene panels covering > 100 genes, WES and whole-genome sequencing (WGS) are becoming more manageable, accessible, and cost-effective [37, 38]. *NPC1* and *NPC2* are currently included in gene panels for infantile cholestatic disease [39], early onset ataxia (EOA) [27], dystonia [38], IEMs [37], organic psychosis, early-onset cognitive decline, hepatosplenomegaly, and developmental delay. A number of studies have reported the successful use of NGS-based methods in identifying previously undiagnosed NP-C cases in at-risk cohorts, particularly among patients with cerebellar ataxia of unclear origin – an extremely heterogeneous clinical population in which genetic diagnoses are notoriously difficult to achieve (Table 2; Additional file 1: Table S1). In a study of 96 patients with unexplained EOA (age at onset < 40 years), targeted high-throughput sequencing of 122 known ataxia genes including *NPC1* and *NPC2*, NP-C diagnoses were confirmed in 2/96 patients (2.1%) [27]. The total frequency of *NPC1/NPC2* gene variants was 8/192 (4.2%), indicating an enrichment of rare *NPC1/NPC2* variants in EOA subjects compared with the general population (203/12,962 (1.6%)). Another study found two (6.1%) NP-C cases in 33 patients with suspected inherited ataxia (age at onset < 50 years) using mini-exome and copy-number variation (CNV) analysis [40]. Using WES, Pyle et al. reported two siblings (5.7%) with NP-C among 22 randomly selected families affected by unexplained ataxias [41]. Castro-Fernández and colleagues identified three patients with previously undiagnosed NP-C among 26 adults with progressive ataxia and other movement disorders, using targeted gene panel sequencing (Sobrido MJ, personal communication).

Liver disease is common early in the course of NP-C, and cohorts of young patients have been assessed using NGS to rule out genetic causes of infantile cholestasis. In independent studies of such patients, McKay et al. [42] and Herbst et al. [43] diagnosed NP-C in 1/228 (0.4%) and 1/6 (16.7%) subjects using custom-designed gene panels targeting *NPC1*/*NPC2* alongside other genes associated with cholestatic disease in infancy and childhood.

#### Blood biomarker screening

Plasma oxysterol assays are now available in over 30 laboratories worldwide, and findings from their use have been reported in a number of screening studies (Table 2; Additional file 2: Table S2). Plasma lysosphingolipid and bile acid assays are relatively new and have the advantage of being detectable in dried blood spots (DBS). However, to date, there are no published reports on their use in NP-C screening.

Two prospective studies that included patients with clinical suspicion of NP-C and which used the oxysterol biomarker, cholestane-3β,5α,6β-triol (C-triol), provided NP-C detection rates of 4.0% [44] and 9.8% [45]. In a further cohort study of patients referred for either cholestasis/hepatosplenomegaly/isolated splenomegaly, or psychomotor regression/retardation, Zhang et al. diagnosed NP-C in 4.0% of patients based on elevated plasma levels of another oxysterol, 7-ketocholesterol (7-KC) [46]. In all three studies, diagnoses were confirmed by genetic analysis of *NPC1/NPC2* mutations.

Other biomarker methods have been variably applied to screen patient cohorts for NP-C. In 236 patients with clinical suspicion of NP-C, De Castro et al. [47] diagnosed 10 patients (4.2%) based on plasma chitotriosidase (ChT) and C-C motif chemokine ligand 18 (CCL18/PARC) levels alongside NP-C SI assessments. Three further NP-C cases were identified in subsequent evaluations of patient family members. In another study of children referred for metabolic testing due to symptoms suggestive of LSDs, Sheth et al. [48] reported four NP-C patients (0.1%) based on filipin staining of cultured fibroblasts. A screening study of 83 patients with unclassified cognitive impairment did not report any NP-C case based on plasma biomarkers (ChT and C-triol), clinical symptoms and NP-C SI [49].

Finally, findings are pending from a further screening study in adults with a first episode of acute psychosis based on a panel of biomarkers and metabolites, where included patients are being screened for a range of IEMs and immunological disorders (CJ Hendriksz, personal communication).

### Studies based on archived (biobank) samples

Biobank studies involve the analysis of historical/archived blood, tissue or genetic materials. Currently there are no published biobank-based screening studies on NP-C, but reports of this study type are expected in the future. Cebolla et al. reported the use of archived biobank plasma samples to evaluate the utility of plasma 7-KC, ChT and CCL18/PARC in 97 patients with NP-C versus a number of control groups [50]. Plasma 7-KC concentration allowed discrimination between NP-C patients, NP-C carriers, and GD patients, but not from patients with NPA/B. Of note, plasma 7-KC and CCL18/PARC in patients with high NP-C SI scores were considered more useful than other biomarkers for defining which patients should undergo confirmatory genetic testing.

### Studies based on medical chart review and bioinformatics/data mining

Screening studies based on patient file and clinical chart review have been conducted in order to detect new NP-C cases as well as to estimate the incidence of NP-C (Table 2; Additional file 3: Table S3). Yerushalmi et al. reviewed clinical and laboratory information from 40 neonates with cholestasis at a paediatric liver centre [51]. Clinical chart review and confirmatory cholesterol esterification assays, liver lipid measurements and genetic analyses identified NP-C in three babies (7.5%) who were initially thought to have idiopathic neonatal hepatitis. Hegarty et al. analysed historical data from clinical and laboratory assessments in 127 newborns and infants with acute liver failure [52], and diagnosed three NP-C patients (2.4%) out of a total of 36 (28.3%) who had a confirmed metabolic aetiology.

Historical health surveillance data can also be accessed to screen for rare conditions. A study from the British National Surveillance Unit (BPSU) provided cross-sectional data on the occurrence of rare disorders including variant Creutzfeldt-Jacob syndrome (vCJD) and NP-C as underlying causes of progressive intellectual and neurological deterioration (PIND) [53, 54]. Over 12 years, 2636 patients aged < 16 years were sent for further assessment of underlying rare disorders, and subsequent expert review of anonymised patient records reached diagnoses that explained observed symptoms in 1114 patients (42%). Among those, NP-C was detected in 38 patients (1.4%). A 2017 update of the study supported the original detection rate (1.3%) [54], and the lifetime risk of NP-C as a cause of PIND among children was estimated at 0.38 per 100,000 live births. Notably, both of these studies highlighted high rates of PIND in areas with higher rates of consanguinity – a recognised predisposing factor in NP-C [15].

Data mining studies, where pre-existing databases are examined to generate new data, represent another form of retrospective, file-based patient screening. A UK study compiled information from regional and national patient registries, reporting a higher prevalence of autosomal recessive conditions (including NP-C) in an ethnic subpopulation (*N* = 13,000) versus the general population [55]. Similar to the BPSU health surveillance studies [53, 54], this finding served to highlight the influence of consanguinity/endogamy on the prevalence of autosomal recessive diseases in some UK communities.

A data mining project employing a bioinformatics methodology is currently underway in Germany. This project, called “*mine*RARE”, aims to identify patients with rare disorders (including NP-C) by using semantic text-mining of electronic medical records. Results are currently pending (T Klopstock, personal communication).

### Studies based on newborn screening

Newborn screening in ultra-rare IEMs, particularly those associated with late-onset symptoms, requires consideration of a number of ethical, clinical, legal and cultural issues [56, 57]. NP-C is not currently included in routine newborn screening programs due to: 1) the vast heterogeneity of clinical manifestations and prognosis; 2) the nature of therapeutic benefits achievable with therapy [57]; and 3) the fact that in many patients (20–30%), symptom onset occurs relatively late in life [58, 59]. Hence, here we use the term ‘newborn screening’ to indicate screening in neonates with clinical abnormalities indicating possible NP-C or other URDs (e.g., perinatal liver disease), and in patients from at-risk clinical groups who did not have observable abnormalities. As such, newborn screening for NP-C can be considered a special subtype of prospective screening studies.

Two studies have investigated the utility of newborn screening for LSDs in general, or NP-C specifically. Pinto et al. reported a 20-year retrospective analysis of 29 different LSDs at a reference centre for antenatal diagnosis [60]. A total of 353 LSD patients were identified out of 4700 cases, among whom 18 patients (0.4%) were diagnosed with NP-C. Based on these results the birth prevalence of NP-C was estimated at 2.2 cases per 100,000. Using oxysterol measures as a screening approach, Polo et al. reported substantially increased levels of both C-triol and 7-KC in 6 out of 7 neonates from a selected cohort with severe cholestasis and suspicion of NP-C [61]. However, genetic testing confirmed a diagnosis of NP-C in only one of these. The observed high rate of false-positives in this cohort was considered a potential pitfall of oxysterol analysis as a screening tool in cholestatic neonates.

## Recommendations on screening set-up for ultra-rare IEMs

There is significant overlap between disease features of NP-C and other ultra-rare IEMs, and similar challenges are faced upon screening for these diseases. Based on published NP-C screening studies and our own experiences, we identified key issues related with likelihood of successful screening and developed a set of recommendations for the setup of screening studies in ultra-rare IEMs (Table 3). General guidelines and local, national and international requirements for good practice in clinical studies also need to be considered.

**Table 3:** Key factors influencing success of screening studies for ultra-rare IEMs

Factor	Recommendation
Team	Ensure patient detection and data quality through use of multidisciplinary investigator teams
Cohort size	Bear in mind that larger cohorts, possibly recruited via expert consortia/registries in at-risk cohorts, help capture the full phenotype range and prevalence data
Inclusion	Consider the impact of inclusion criteria that are neither too restrictive nor too broad
Methods	Employ methods based on associated advantages/limitations, minimally invasive sampling, formal requirements, and possible confounding factors
Genetics	Consider that large NGS gene panels/WES allow screening for multiple diseases in whole cohorts, and factor in the sensitivity and specificity of genetic profiling methods
Biomarkers	Choose biomarkers bearing in mind their sensitivity, specificity, validation, sample stability and ease of transport, and assay turnaround times
Clinical assessment	Use available simple clinical tools that allow quick analyses of relevant symptom clusters
Laboratories	Select reference laboratories with well-established infrastructure for selected, validated diagnostic method(s)
Consent	Take patient consent limits into account, particularly for retrospective chart reviews/biobanks
Sustainability	Preserve awareness and knowledge from screening studies in local diagnostic procedures and/or follow-up processes
Increased awareness	Raise awareness of rare disorders as a group represent a significant healthcare problem: this can aid referral to appropriate specialist clinics in time

The main objectives of ultra-rare IEM screening programs are to: 1) identify patients who would otherwise go undetected or receive a delayed diagnosis and thus go deprived of proper treatment; 2) characterize the phenotypic range where current suspicion is only based on a classical clinical syndrome; 3) evaluate gene variants as possible contributors to other diseases; and, 4) improve disease awareness to ensure inclusion of rare disorders in differential diagnosis. Additional deliverables from screening for ultra-rare IEMs include; assessment of gene variant effects in heterozygotes (carriers); identification of other unrecognised diseases during differential diagnosis; estimation of disease prevalence and incidence.

### Study design

Key factors that should be addressed in designing a screening study include: 1) identification and selection of an appropriate patient cohort based on available data (e.g., from living patients, biobank samples, medical charts); 2) the use of optimal and accepted diagnostic method(s) (see *Diagnostic methods*); and 3) relevant local factors (e.g., available expertise, funding, regulations).

- Consider which at-risk patient populations might include ‘hidden’ ultra-rare IEM patients.
- Define simple, concise screening objectives addressing appropriate clinical disease phenotypes.
- Involve the lay-community through medical education on IEM natural history (as for NP-C).

#### Prospective study designs

Prospective studies have the advantage of allowing further examination in suspected patients. However, in the case of ultra-rare IEMs, prospective studies can pose great challenges for patient recruitment and/or require prolonged observation periods in order to confirm a diagnosis.

#### Retrospective study designs

Retrospective studies are more suited to patient chart reviews and biobank analyses, and generally have simpler requirements versus prospective studies regarding logistics and planning. However, retrospective studies in ultra-rare IEMs may be prone to bias due to limited patient follow-up. Retrospective studies also depend on analyte stability, and expiration of patient consent may be a limiting issue. Limitations on data accuracy/completeness, potential for recall bias, and existence of missing data can be encountered in medical chart reviews. Access to corresponding physicians and/or patients (e.g., outdated contact details, patient death, physician retirement) also affect findings when older files or biobank samples are included. Biobanks must allow proper pre-selection of at-risk patients. Care must be taken to avoid over-interpretation of retrospective data, especially when information at hand is incomplete.

### Patient population

Direct access to target screening population must be ensured.

- Effective collaboration between general physicians and expert centres is crucial in ultra-rare IEM screening programs, as general physicians are usually closer to the patients and their main healthcare reference.
- Common scenarios for patient sourcing include: at-risk cohorts in patients with key ultra-rare IEM symptoms; verification of published serendipitous findings in specific patient subgroups; patients considered at risk for scientific reasons (e.g., similar brain pathology in in neurodegenerative disorders).

### Cohort size

Formal guidance on appropriate cohort sizes is lacking for many diseases, particularly ultra-rare IEMs. Target patient numbers should be addressed in a pragmatic manner according to the study design, diagnostic methods and epidemiological information. Larger screening cohorts potentially capture more disease phenotypes and provide more accurate prevalence estimates, but demand more resources and bear a higher chance of false positives.

- The number of potential patients affected by an ultra-rare IEM is very small. Hence, studies may need to include multiple centres or involve pertinent disease consortia or registries (e.g., the autosomal-recessive ataxia consortium, ‘PREPARE’ and the EOA registry in the case of NP-C).
- The availability of historical data should be considered in studies aiming to estimate disease prevalence or incidence.
- Relevant age groups/disease stages are important where early identification is required in an ultra-rare IEM.
- Endogamy and consanguinity must be considered when studying IEMs in certain regions.

### Inclusion/exclusion criteria

Clear and easy-to-follow inclusion/exclusion criteria should be defined that meet the consensus of the scientific community. The restrictiveness of chosen criteria influences detection accuracy: broader inclusion typically results in low detection rates, whereas more stringent criteria give higher detection rates. While this may seem obvious it has a particularly high impact in ultra-rare IEMs.

- Cohort inclusion and exclusion criteria (e.g., based on symptom severity or comorbidities) should be defined clearly for specific at-risk patient groups in ultra-rare IEMs that feature high phenotypic heterogeneity.
- Overly specific criteria might miss mild/atypical patients, which are common in ultra-rare IEMs.
- Overly specific criteria might miss patients with mild/atypical symptoms, which are common in ultra-rare IEMs. Furthermore, a bias toward subjects with classical disease presentations is likely present in the published literature, and thus the full phenotypic spectrum of rare disorders may not be well known.

### Diagnostic methods

Screening methods for inherited disorders typically include clinical assessments of specific disease symptoms, biomarkers, and genetic tests. All three of these methods have utility for the detection of patients when applied on a broad scale. Taking NP-C as an example, key features of these methods are summarised in Table 4.

- Multi-analyte MS/MS biomarker panels or large NGS gene panels/WES allow cost-effective, simultaneous screening for diseases associated with clinical features that are common within a chosen screening cohort: such techniques can currently be applied in DBS samples for over 30 IEMs and are of particular use in newborn screening.
- DBS samples are particularly convenient in terms of storage and transport.
- Gene panels should cover all known diseases that can cause the same manifestations.

**Table 4:** Key features of diagnostic methods for ultra-rare IEMs: NP-C as an example

Method	Examples for NP-C	Key features
Biomarkers	• Oxysterols (C-triol, 7-KC)	• Advantages
	• Lysosphingolipids (Lyso-SM-509, lysosphingomyelin)	• Objective, quantitative methodology
		• Rapid, practical and cost-effective*
	• Bile acids (3β,5α,6β-trihydroxycholanic acid)	• Biomaterials easily accessible and transportable
		• Disadvantages
		• Available for relatively few ultra-rare IEMs
		• Requires that disease of interest is already in differential diagnosis
		• Patient heterogeneity can present a hurdle, with possible false-negatives/positives
Genetic analysis	• Single-gene sequencing	• Advantages
	• Gene panel (e.g., ataxia panel)	• Objective screening data
	• WES	• No requirement for differential diagnosis
	• WGS	• Can provide information on diseases not in differential diagnosis
		• Might indicate alternate molecular diagnosis
		• Disadvantages
		• Not yet widely available without appreciable costs
		• Limited information on pathogenicity of unique gene variants (potential false negatives and false positives)
		• Challenging management of VUS in symptomatic patients without biochemical marker findings
		• Management of incidental findings
Clinical assessment	• Multi-disciplinary assessment of clinical manifestations	• Advantages
		• Widespread availability of professionals capable of carrying out clinical assessment
	• Differential diagnosis	
	• Assessment of clinical picture from patient files	• Traditional approach set up in healthcare systems
		• Disadvantages
		• Can be time consuming
	• NP-C SI	• Require multiple inter-disciplinary referrals
		• Variation in quality: assessments not based on validated clinical tools require IEM expert knowledge to detect disease
		• Non detection of atypical/non-standard or early-stage presentations due to non-specific clinical phenotypes
		• Do not deliver diagnosis per se although diagnoses can be confirmed using biomarkers and/or genetic methods

^a^Cost effectiveness depending on local infrastructure and/or geographical region; *7-KC* 7-ketocholesterol, *C-triol* cholestane-3β,5α,6β-triol, *NP-C SI* NP-C suspicion index, *VUS* variant of unknown significance, *WES* whole-exome sequencing, *WGS* whole-genome sequencing

The most appropriate diagnostic methods should be chosen based on the following criteria:

1. Quality: the sensitivity, specificity, validity and robustness of the chosen test(s), and methods for identifying false positives and false negatives should be ensured. Methods should be acknowledged by the scientific community and acceptable to patients: the less invasive the better. Diagnostic methods in ultra-rare IEMs are not always supported by substantial published evidence, but may nevertheless be accepted by experts.
2. Suitability: diagnostic reference laboratories need to be experienced with the selected diagnostic method(s), and local infrastructure should grant access to IEM patients and sample shipment in less densely-populated areas.
3. Applicability and ease of use: processing limitations can prevent use of certain methods in some geographical areas, and possible confounding factors (e.g., auto-oxidation in plasma samples) should be taken into account. The effects of local cultural factors on patient agreement to participate must be considered in ultra-rare IEMs: the genetic nature of these disorders requires DNA analysis. The supply of relevant clinical background for less well known ultra-rare IEMs is vital to help diagnostic laboratories interpret findings.

#### Multi-level diagnostic approaches

Combinations of diagnostic methods including clinical assessments, biomarker assays, and/or genetic techniques can reduce the likelihood of screening errors, which is important in uncertain cases, as often seen in ultra-rare IEMs.

- Clinical tools assessing relevant symptom clusters can help distinguish affected patients from the general clinical population and non-affected patients.
- Biomarker analyses typically include confirmation of initial biomarker-identified cases though genetic analysis.
- Genetic screening studies are usually more successful when performed on patients/cohorts that have been selected through clinical assessments and/or biomarker analysis.

### Ethical and regulatory requirements

As in any disease, ethical and regulatory requirements must always be met in ultra-rare IEM screening, and include specific institutional/regulatory ethical approvals, regulatory body expectations, patient consent requirements, Good Clinical Practice (ICH-GCP) standards, and Good Laboratory Practice criteria.

- Control of patients’ personal information is particularly important in ultra-rare IEMs as patients can more easily be identified based on relatively few generic personal data. Thorough data anonymization should be implemented.
- Screening studies for diseases with available targeted therapies should be given priority.

### Screening logistics

Logistics for sample handling, labelling, stability, and transport are crucial in ultra-rare IEMs as diagnostic tests are frequently carried out in specialist laboratories that may not be local. Definition of all aspects of sample storage is also important in biobank-based studies.

### Study team and disease experience

Ultra-rare IEM screening studies are usually conducted by physicians with access to relevant cohorts but not necessarily with relevant expertise. Referring physicians should be well instructed and trained on the key disease signs and symptoms of ultra-rare IEMs.

- Broad experience within the study team is vital: patient detection and data quality is best optimized in a multidisciplinary setup.

### Study legacy or ‘halo’ effects

Potential long-term post-study benefits (‘halo’ effects) should be considered before starting a screening study, especially with less well recognized diseases like ultra-rare IEMs. Examples include: establishment of collaborative structures and improved lines of referral; creation of multi-analyte biomarker or gene panels that can be included in routine practice; and enduring local use of diagnostic methods/algorithms.

- Some ultra-rare IEM screening studies identify very few or no patients during the study observation period but cases can be identified subsequently due to increased local awareness, health provider acceptance of new biomarkers, and establishment of multidisciplinary care networks.
- Raised awareness is a potent factor in considering IEM diagnoses in some cohorts, and has been shown to ease acceptance of biomarker methods.

## Possible future screening strategies in NP-C

A large proportion of published NP-C screening studies have employed combinations of both established and new diagnostic methods. Such strategies may reduce the likelihood of screening errors in the future. A typical diagnostic tactic for NP-C screening would comprise initial clinical examination (e.g., using the NP-C SI) followed by biomarker measurements and genetic validation. Combined approaches like this limit burden to patients and allow a more efficient and cost-saving study set-up [26, 47].

The general consensus among experts involved in NP-C care is that genetic analysis is mandatory for the confirmation of diagnosis [62]. New, rapid genetic sequencing methods such as WES and WGS are likely to allow wider screening across known at-risk patient cohorts in the near future. The potential application of NGS methods as the initial (first-line) diagnostic test in an ultra-rare IEM depends on available resources, genetic mutation types and complexity, disease awareness, and the nature of the disease and patients/cohorts in question. For instance, genetic analysis of *FMR1* variants would not work as a screening method for Fragile X syndrome. Nevertheless, based on experience to date in NP-C, the potential inclusion of ultra-rare IEM genes in large NGS gene panels holds great promise for future screening protocols. The use of WES and WGS databases is growing, and ultra-rare IEM gene databases are increasingly becoming interconnected and/or made public. Where possible, an ‘exome-first’ approach, where WES is conducted as a first step to identify potential new cases in at-risk cohorts, may provide a more direct route to NP-C diagnosis [63]. Such approaches are already being implemented in some centres.

Updated international recommendations for the diagnosis and screening of NP-C classify new biomarker assays alongside genetic analyses as first-line diagnostic methods, and note that most diagnoses can be confirmed by the combined use of these methods [23]. The prospect of automatically linking large registries for at-risk clinical cohorts to relevant biomarker analyses is an interesting prospect for improving the detection of further cases, but is currently only applicable in the academic research setting. Such an approach is currently being assessed for linking lysosphingolipid assays with the EOA Registry in Germany (M Synofzik, personal communication).

## Conclusions

Screening studies in NP-C, which is considered as a suitable role model for ultra-rare IEMs in general, are associated with a number of challenges related to the ultra-rare nature of the disease. To date, screening for NP-C has largely been based on single-patient studies, small case series, and targeted cohort studies in at-risk patient groups. However, the emergence of new diagnostic methods over the last 5–10 years has provided opportunities to screen for NP-C on a larger scale in whole at-risk cohorts [64–66].

NP-C is difficult to detect using routine methods as it is a lysosomal disorder that is not detected by standard enzyme panels. The advent of readily available, specific blood biomarkers has largely overcome this limitation, and the inclusion of specific biomarker assays into metabolic screening panels that can easily be applied in suspected patients or cohorts is now achievable. At a number of centres, biomarker assays have been used as a first-line step in diagnosing NP-C, allowing an exponential increase in the number of patients that can be screened in a short time [23, 26]. However, the position of biomarker testing in the diagnostic pathway varies between centres.

Broad genetic screening of patients with symptoms of unclear origin using NGS gene panels can now be conducted in large patient populations as well as in individual patients with no clear molecular diagnosis, and the use of NGS is expected to grow significantly in the future. Gene panels allow diagnostic testing for multiple ultra-rare IEMs. A growing number of centres are adopting an ‘exome-first’ diagnostic work-up in their routine practice (e.g. in Nijmegen, the Netherlands and Tübingen, Germany), whereby WES is applied widely before more detailed laboratory work-up.

New, simple digital clinical screening tools that allow rapid analyses of relevant symptom clusters are increasingly becoming available [15, 20]. As an example, the NP-C SI allows rapid appraisal of the likelihood of NP-C at initial patient presentation or soon after, and helps to direct further, more detailed confirmatory tests.

Combining clinical, biomarker and genetic diagnostic methods represents the most effective way to identify new NP-C cases. Updated diagnostic and screening recommendations for NP-C have been developed that cover all available diagnostic methods, and should be considered when formulating any new screening study [11, 23].

Overall, the value of screening for ultra-rare IEMs such as NP-C represents a trade-off between funding costs on one hand, and benefits from targeted therapy in what are usually quite small yields of previously unidentified patients on the other [29]. In terms of cost-effectiveness, two types of study design can now be considered: a) based on gene panels and/or multi-analyte biomarker panels, which is associated with higher initial costs but can cover a large number of diseases [27, 42, 43]; and b) using relatively low-cost plasma- or DBS-based biomarkers that cover only single or a few diseases [67]. There is also an asymmetry in the number of available studies and resources devoted to disease screening for different IEMs based on the commercially-funded availability of targeted therapies. While this might potentially introduce some bias to reported case identification, this should not impede analysis of the existing literature and extraction of useful lessons.

Many of the learnings from NP-C screening studies can be extrapolated to other ultra-rare IEMs due to similarities in a number of key disease factors. These recommendations can therefore serve as a guide for planning patient screenings in ultra-rare IEMs in general.

## Additional files

Additional file 1: Summary of published screening studies based primarily on genetic analysis. (DOCX 27 kb)

Additional file 2: Summary of published screening studies based primarily on biomarker analysis. (DOCX 29 kb)

Additional file 3: Summary of published screening studies based primarily on medical chart review and bioinformatics/data mining. (DOCX 29 kb)

## Abbreviations

7-KC: 7-ketocholesterol
ChT: Chitotriosidase
CNS: Central nervous system
C-triol: Cholestane-3β,5α,6β-triol
DBS: Dried blood spot
EOA: Early-onset ataxia
GC/MS: Gas chromatography-mass spectrometry
GD: Gaucher disease
IEMs: Inborn errors of metabolism
LSD: lysosomal storage disease
MCADD: Medium-chain acyl-CoA dehydrogenase deficiency
MEGDEL: 3-methylglutaconic aciduria with deafness, encephalopathy and Leigh-like
MPS: Mucopolysaccharidoses
NP-A/NP-B: Niemann-Pick types A and B
NP-C SI: NP-C suspicion Index
NP-C: Niemann-Pick disease type C
PCR: Polymerase chain reaction
PIND: Progressive intellectual and neurological deterioration
PKU: Phenylketonuria
URDs: Ultra-rare diseases
vCJD: variant Creutzfeldt-Jacob syndrome.
