# Supplementary material for: Recommendations for patient screening in ultra-rare inherited metabolic diseases: what have we learned from Niemann-Pick disease type C?
Source: Orphanet J Rare Dis. 2019 Jan 21;14:20. doi: 10.1186/s13023-018-0985-1 (PMC6341610; doi:10.1186/s13023-018-0985-1)
Supplement: Supplementary file 1 — Summary of published screening studies based primarily on genetic analysis. (DOCX 27 kb) [file 13023_2018_985_MOESM1_ESM.docx]

# Additional file 1: Table S1. Summary of published screening studies based primarily on genetic analysis

| **Cohort (reference)** | **Study population** | **Design / observation period** | **Centres / countries** | **Screening method(s)** | **Patients identified, n/N (%)*** |
| --- | --- | --- | --- | --- | --- |
| Pinto et al. 2004 [55] | Antenatal patients with suspected LSD based on metabolic screening N = 353 | Retrospective observational /  20 years | Single centre /  Portugal | *NPC1/NPC2* sequencing^†^ Enzyme assay panel Filipin staining | Patients: 18 (0.4%) |
| Bauer et al. 2013 [33] | Adults with neurological and psychiatric symptoms N = 250 | Prospective observational /  No period specified | Multicentre / EU and US | *NPC1/NPC2* sequencing ^†^ | Patients: 3 (1.2%)  Carriers: 12 (4.8%) |
| Schicks et al. 2013 [34] | Adults aged <40 years with ataxia, EOCD and medical history suggesting recessive disease M = 24 | Prospective observational / 4 years | Single centre / Germany | *NPC1/NPC2* sequencing^†^ Filipin staining | Patients: 4 (16.7%) |
| Zech et al. 2013 [35] | Adults with PD (n = 563), FTD (n = 133) or PSP (n = 94) | Prospective controlled observational  No period specified | Multicentre /  Germany | *NPC1/NPC2* sequencing^†^ Biomarkers | Patients: 0 (0%) Carriers (PD): 6 (1.1%) |
| McKay et al. 2014 [41] | Infants with jaundice/cholestasis N = 228 | Prospective observational / No period specified | Multicentre / UK | *NPC1/NPC2 NGS sequencing* (WES) in gene panel | Patients: 1 (0.4%) Carriers: 5 (2.2%) |
| Herbst et al. 2015 [42] | Infants with jaundice/cholestasis  N = 6 | Prospective observational / No period specified | Single-centre /  Germany | *NPC1/NPC2 NGS sequencing* (WES) in gene panel | Patients: 1 (16.7%) |
| Synofzik et al. 2015 [27] | Adolescents/adults with unexplained early-onset ataxia N = 96 | Prospective observational / 6 years | Multicentre /  Germany | Targeted high-throughput *NPC1/NPC2* sequencing | Patients: 2 (2.1%) |
| Pyle et al. 2015 [40] | Patients with unexplained inherited, sporadic ataxias N = 35 | Prospective observational /  No period specified | Single centre /  UK | *NPC1/NPC2* NGS sequencing (WES) | Patients: 2 (5.7%) |
| Marelli et al. 2016 [39] | Adolescents/adults with probable early-onset ataxia N = 33 | Prospective observational / No period specified | Multicentre / France | Mini-exome/CNV-based NGS gene panel | Patients: 2 (6.1%) |
| Cupidi et al. 2017 [38] | Adults with early-onset dementia-plus N = 50 | Prospective observational /  No period specified | Single centre / Italy | *NPC1/NPC2* sequencing^†^ NP-C SI | Patients: 0 (0%) Carriers: 4 (8.0%) |
| Nanetti et al. 2017 [36] | Adults with suspected HD  N = 18 | Prospective observational /  No period specified | Single centre / Italy | *NPC1/NPC2* sequencing^†^ | Patients: 3 (16.7%) |
| Topcu et al. 2017 [37] | Family members of NPC1/NPC2 probands  N = 510 | Prospective observational /  No period specified | Single centre / Turkey | *NPC1/NPC2* sequencing^†^ | Patients: 2 (0.4%)  Carriers: 116 (22.7%) |
| Mavridou et al. 2014 [43] | Family members of two NP-C patients  N = 153 | Retrospective observational /  No period specified | Single centre / Greece | *NPC1/NPC2* sequencing^†^ | Patients: 0 (0%) Carriers: 64 (41.8%) |

**n/N (%), number of cases detected per cohort or study over total number of subjects in cohort/study (% based on n/N); ^†^Sanger sequencing; CNV, copy-number variation; FTD, frontotemporal dementia; LSD, lysosomal storage disease; NGS, next-generation sequencing; PD, Parkinson's disease; PSP, progressive supranuclear gaze palsy; SI, suspicion index; WES, whole-exome sequencing; HD, Huntington´s disease.*
