# Supplementary material for: Recommendations for patient screening in ultra-rare inherited metabolic diseases: what have we learned from Niemann-Pick disease type C?
Source: Orphanet J Rare Dis. 2019 Jan 21;14:20. doi: 10.1186/s13023-018-0985-1 (PMC6341610; doi:10.1186/s13023-018-0985-1)
Supplement: Supplementary file 2 — Summary of published screening studies based primarily on biomarker analysis. (DOCX 29 kb) [file 13023_2018_985_MOESM2_ESM.docx]

# Additional file 2: Table S2. Summary of published screening studies based primarily on biomarker analysis

| **Reference** | **Study population** | **Design / observation period** | **Centres / countries** | **Screening method(s)** | **Patients identified, n/N (%)*** |
| --- | --- | --- | --- | --- | --- |
| Zhang et al. 2014 [46] | Children and adults with cholestasis/HSL or psychomotor regression/retardation N = 302 | Prospective observational /  2 years | Single centre / China | Oxysterol level (7-KC) *NPC1/NPC2* sequencing^†^ | Patients: 12 (4.0%) |
| Sheth et al. 2014 [48] | Patients with clinical features suggesting LSDs N = 1,110 | Prospective observational /  10 years | Multicentre/ International | Urine/plasma metabolic screen  Filipin staining | Patients: 4 (0.4%) |
| Cebolla et al. 2015 [49] | Patients with NP-C  N = 97 | Retrospective observational /  NA | Single centre / Spain | Oxysterol level (7-KC)  ChT, CCL18/PARC  NP-C SI | Patients: NA |
| Reunert et al. 2016 [44] | Patients with suspected NP-C N = 1,800 | Prospective observational / 3 years | Single-centre / Germany | Oxysterol level (C-triol) *NPC1/NPC2* sequencing^†^ | Patients: 72 (4.0%)  Carriers: 24 (1.3%) |
| Ribas et al. 2016 [45] | Patients with suspected NP-C N = 122 | Prospective observational / No period specified | Multicentre/ Brazil | Oxysterol level (C-triol) ChT Filipin staining | Patients: 12 (9.8%) |
| Polo et al. 2016 [56] | Neonates with cholestasis N = 7 | Prospective observational / No period specified | Multicentre /  Italy | Oxysterol levels (7-KC, C-triol) | Patients: 1 (14.0%) |
| De Castro et al. 2017 [47] | Patients with ≥2 symptoms typically seen in NP-C N = 236 | Prospective observational / 2 years | Multicentre / Spain | ChT, CCL18/PARC NP-C SI *NPC1/NPC2* sequencing^†^ | Patients: 10 (4.2%) |

**n/N (%), number of cases detected per cohort or study over the total number of subjects in cohort/study (% based on n/N); ^†^Sanger sequencing; HSL, hepatosplenomegaly; LSD, lysosomal storage disease; NA, not applicable; NR, not reported; SI, suspicion index.*
