# Supplementary material for: Recommendations for patient screening in ultra-rare inherited metabolic diseases: what have we learned from Niemann-Pick disease type C?
Source: Orphanet J Rare Dis. 2019 Jan 21;14:20. doi: 10.1186/s13023-018-0985-1 (PMC6341610; doi:10.1186/s13023-018-0985-1)
Supplement: Supplementary file 3 — Summary of published screening studies based primarily on medical chart review and bioinformatics/data mining. (DOCX 29 kb) [file 13023_2018_985_MOESM3_ESM.docx]

# Additional file 3: Table S3. Summary of published screening studies based primarily on medical chart review and bioinformatics/data mining

| **Cohort (reference)** | **Study population** | **Design / observation period** | **Centres / countries** | **Screening method(s)** | **Patients identified, n/N (%)*** |
| --- | --- | --- | --- | --- | --- |
| Yerushalmi et al. 2002 [50] | Neonates with jaundice/cholestasis N = 40 | Retrospective, observational /  2 years | Single centre / US | Medical chart review *NPC1/NPC2* sequencing^†^ Other laboratory tests^‡^ | Patients: 3 (7.5%) |
| Verity et al. 2010 [52] | Children (<16 years) with suspected early-onset cognitive impairment N = 2,636 | Prospective surveillance /  12 years | Multicentre / UK | Clinical case surveillance reporting | Patients: 38 (1.4%) |
| Hegarty et al. 2015 [51] | Patients <5 years old with acute liver failure N = 127 | Retrospective observational /  10 years | Single centre /  UK | Clinical, laboratory, and outcome data | Patients: 3 (2.4%) |
| Wassif et al. 2016 [14] | Subjects (all ages) included in 4 large, sequencing projects N = 17,754 chromosomes | Retrospective genetic database / No period specified | Multicentre /  International | Historical *NPC1/NPC2* sequencing (*WES*) | Patients: 0 (0%) (1 in 89,229^††^) |
| Winstone et al. 2017 [53] | Children aged < 16 years with progressive intellectual and neurological deterioration  N = 3,979 | Retrospective cross-sectional national surveillance  18 years | Multicentre / UK | Clinical case surveillance reporting | Patients: 53 (1.3%) |
| Corry 2014 [54] | UK ethnic subjects with suspected autosomal recessive conditions  N = >13,000 | Retrospective analysis of regional and national patient registries  3 years | Multicentre / UK | Clinical case surveillance reporting | NR |

**n/N (%), number of cases detected per cohort or study over total number of subjects in cohort/study (% based on n/N); ^†^Sanger sequencing; ^‡^other laboratory tests including, but not limited to filipin staining, cholesterol esterification assay, liver/skin biopsy and electron microscopy; ^††^Calculated frequency of pathogenic NPC1/NPC2 variants; IEM, inborn error of metabolism; WES, whole-exome sequencing.*
